# Theoretical Tinnitus Framework: A Neurofunctional Model

**DOI:** 10.3389/fnins.2016.00370

**Published:** 2016-08-19

**Authors:** Iman Ghodratitoostani, Yossi Zana, Alexandre C. B. Delbem, Siamak S. Sani, Hamed Ekhtiari, Tanit G. Sanchez

**Affiliations:** ^1^Neurocognitive Engineering Laboratory, Institute of Mathematics and Computer Sciences, University of São PauloSão Carlos, Brazil; ^2^Center of Mathematics, Computation and Cognition, Federal University of ABCSão Bernardo do Campo, Brazil; ^3^Institute of Mathematics and Computer Sciences, University of São PauloSão Carlos, Brazil; ^4^WHO Research- World Hearing OrganizationSan Jose, CA, USA; ^5^Iranian National Center for Addiction Studies, Tehran University of Medical SciencesTehran, Iran; ^6^ENT Department, Faculty of Medicine, University of São PauloSão Carlos, Brazil; ^7^Instituto Ganz SanchezSão Paulo, Brazil

**Keywords:** tinnitus modeling, cognitive processes in tinnitus, attention role in tinnitus, tinnitus brain network, evaluation learning role in tinnitus

## Abstract

Subjective tinnitus is the conscious (attended) awareness perception of sound in the absence of an external source and can be classified as an auditory phantom perception. Earlier literature establishes three distinct states of conscious perception as unattended, attended, and attended awareness conscious perception. The current tinnitus development models depend on the role of external events congruently paired with the causal physical events that precipitate the phantom perception. We propose a novel Neurofunctional Tinnitus Model to indicate that the conscious (attended) awareness perception of phantom sound is essential in activating the cognitive-emotional value. The cognitive-emotional value plays a crucial role in governing attention allocation as well as developing annoyance within tinnitus clinical distress. Structurally, the Neurofunctional Tinnitus Model includes the peripheral auditory system, the thalamus, the limbic system, brainstem, basal ganglia, striatum, and the auditory along with prefrontal cortices. Functionally, we assume the model includes presence of continuous or intermittent abnormal signals at the peripheral auditory system or midbrain auditory paths. Depending on the availability of attentional resources, the signals may or may not be perceived. The cognitive valuation process strengthens the lateral-inhibition and noise canceling mechanisms in the mid-brain, which leads to the cessation of sound perception and renders the signal evaluation irrelevant. However, the “sourceless” sound is eventually perceived and can be cognitively interpreted as suspicious or an indication of a disease in which the cortical top-down processes weaken the noise canceling effects. This results in an increase in cognitive and emotional negative reactions such as depression and anxiety. The negative or positive cognitive-emotional feedbacks within the top-down approach may have no relation to the previous experience of the patients. They can also be associated with aversive stimuli similar to abnormal neural activity in generating the phantom sound. Cognitive and emotional reactions depend on general personality biases toward evaluative conditioning combined with a cognitive-emotional negative appraisal of stimuli such as the case of people with present hypochondria. We acknowledge that the projected Neurofunctional Tinnitus Model does not cover all tinnitus variations and patients. To support our model, we present evidence from several studies using neuroimaging, electrophysiology, brain lesion, and behavioral techniques.

## Introduction

Tinnitus has been described as the conscious perception of sounds, usually hissing or ringing, in the absence of an external sound source. It can be a significant annoyance and can noticeably decrease the quality of life. Statistically, most of the people who suffer from tinnitus tend to live with the condition without seeking any treatment. Tinnitus affects 30% of the general population, mostly affecting the elderly population. 6% have debilitating symptoms (Heller, 2003), and an equal 6% prevalence has also been found in children (Mills et al., 1986; Coelho et al., 2007; Savastano, 2007).

In many cases, tinnitus is a serious condition that becomes a chronic problem and is often reported as “annoying” and “severely affecting quality of life.” It has been demonstrated that 60% of tinnitus patients suffered from lifetime and 55% suffered from current psychiatric disorders, while depression and anxiety were the most common types of comorbidity disorders (Malakouti et al., 2011). Over the past few decades, several hypotheses have attempted to explain tinnitus. A few of them have reached animal and human trials and successfully received acceptance by the academic and clinical communities and reached diagnosis and/or rehabilitation product stages.

Several animal models have attempted to explain tinnitus by revealing its physiological characteristics in different processing centers of the auditory system. In the dorsal cochlear nucleus (DCN), enhanced firing rates were distinguished after intense acoustic exposure (Kaltenbach et al., 1998; Brozoski et al., 2002; Chang et al., 2002). In the inferior colliculus, elevated firing rates were distinguished after large doses of salicylate induction (Jastreboff and Sasaki, 1986; Chen and Jastreboff, 1995). In addition, it has been established that noise trauma generates hyperactivity in the auditory cortex (Eggermont and Komiya, 2000; Seki and Eggermont, 2003; Zhang et al., 2011). Other tinnitus-related animal studies include the following, tonotopic reorganization in auditory cortex (Eggermont and Roberts, 2004; Eggermont, 2006; Stolzberg et al., 2011), increase in spontaneous activity in DCN and the inferior colliculus (Kaltenbach and McCaslin, 1996; Zhang and Kaltenbach, 1998; Noreña and Eggermont, 2003), magnification in auditory central gain (Sun et al., 2009; Zeng, 2013; Auerbach et al., 2014), and synchronization of neuronal activities (Strauss et al., 2005, 2008; Dominguez et al., 2006; Lorenz et al., 2009). Moreover, a recent imaging study also demonstrated that different inhibitory and excitatory neurotransmitters modulate the tinnitus-dependent hyperactivity (Middleton et al., 2011).

Furthermore, neuroanatomical and activation alteration of the auditory pathways were correlated with abnormal activities in the non-auditory brain areas in tinnitus patients vs. control volunteers, specifically by means of Magnetic Resonance Imaging (MRI) (Crönlein et al., 2007; Langguth et al., 2007; Landgrebe et al., 2009; Schneider et al., 2009; Husain et al., 2011; Boyen et al., 2013), Functional MRI (Smits et al., 2007; Lanting et al., 2008), Positron Emission Tomography (PET) (Arnold et al., 1996; Lockwood et al., 1998; Andersson et al., 2000; Langguth et al., 2006; Plewnia et al., 2007), Single-Photon Emission Computerized Tomography (SPECT), (Gardner et al., 2002; Marcondes et al., 2006), and Magnetoencephalography (MEG) (Mühlnickel et al., 1998).

Integrating information about tinnitus from different studies can provide general tinnitus models (Jastreboff et al., 1988b; Jastreboff and Hazell, 2004; Tyler et al., 2008; Rauschecker et al., 2010). Jastreboff et al. (1988a) proposed the neurophysiological model of tinnitus (NM), which resulted in a tinnitus management procedure known as Tinnitus Retraining Therapy. NM differs radically from previous models due to the following postulations: (a) Tinnitus is a phantom auditory perception (Jastreboff, 1990); (b) Tinnitus occurs due to the interaction of different brain networks with auditory pathways that result in the conscious perception of the phantom sound. Principally, the limbic system is responsible for growth of tinnitus annoyance (Jastreboff, 1990); (c) Perception of tinnitus is not necessarily the key element that causes tinnitus to be problematic, and it is possible to have reactions to the tinnitus signal without perceiving it (Jastreboff and Jastreboff, 2006); (d) Sustained over-activation of the sympathetic autonomic nervous system is largely responsible for the behavioral manifestation of tinnitus-induced problems (Jastreboff and Jastreboff, 2006); and (e) Once habituation of reactions is sufficiently advanced and the tinnitus signal becomes neutral and unimportant, the habituation of perception follows automatically (Jastreboff and Jastreboff, 2006).

**Figure 1:**
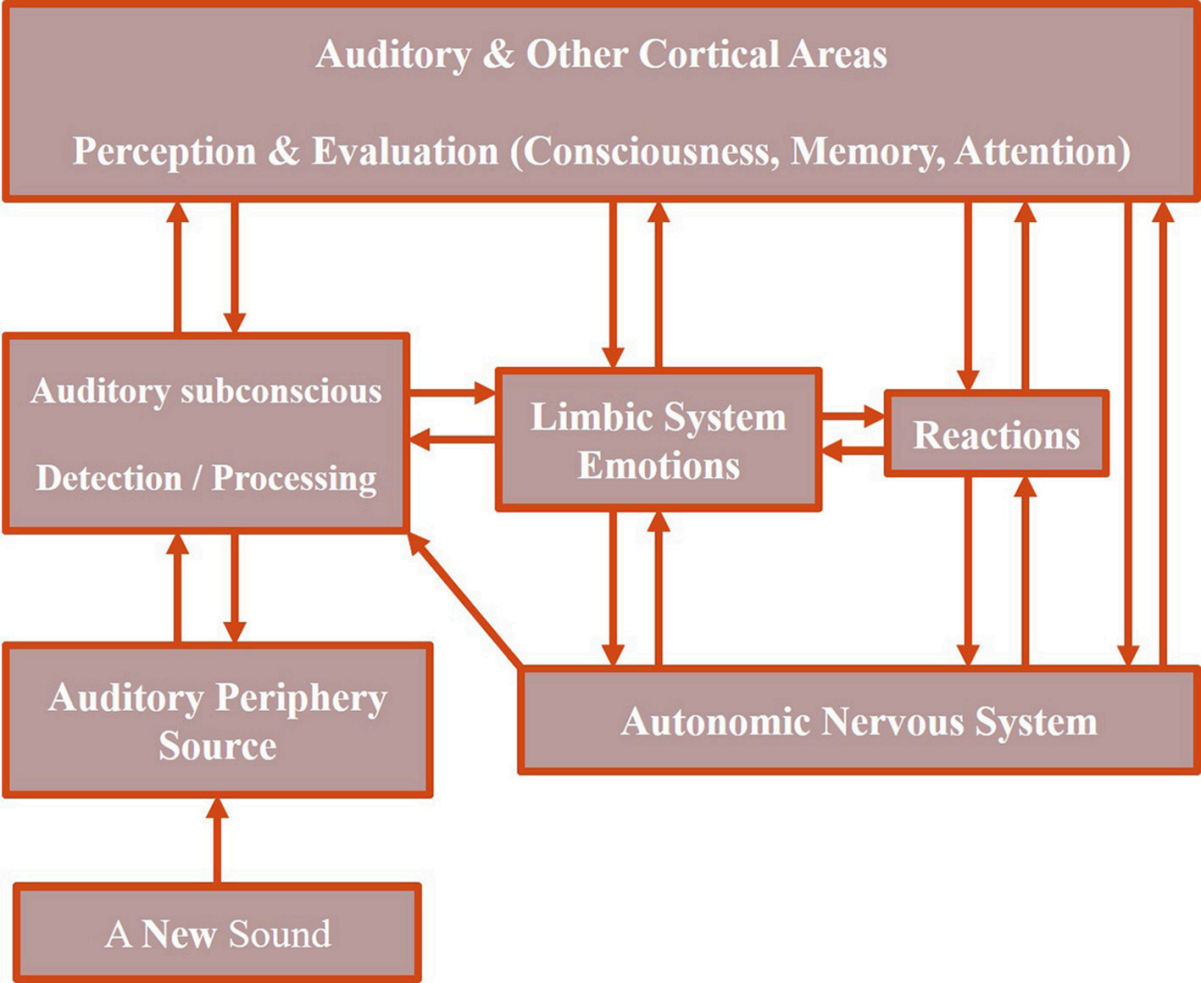
**Neurophysiological model of tinnitus. Adapted from Jastreboff and Jastreboff (2006)**.

NM has considered the involvement of several areas of the central nervous system as well as the autonomous nervous system. The flow of information commonly starts with sound wave stimulation of the peripheral auditory system. The next stage is a two-way connection with the “Auditory Subconscious” regions. The following stage, via a two-way connection, is the “Auditory and Other Cortical Areas” process perception and evaluation of sound and includes functions such as consciousness, memory, and attention. The last neuroanatomical and functional component in NM is the “Limbic System,” which is illustrated in Figure 1. The “Limbic System” is connected via a two-way link to components of “Auditory Subconscious” and “Auditory & Other Cortical Areas.” It is also connected to the “Autonomic Nervous System,” influencing neuroendocrine and autonomic reactions, such as respiratory, circulatory, digestive and hormonal. The “Autonomic Nervous System” sends inputs to the “Auditory Subconscious” component as well as sends and receives inputs from the “Auditory & Other Cortical Areas.” The sixth and the last component is termed “Reactions”, which refers to clinical observations such as annoyance, anxiety, panic, sleep, and concentration disturbances. These “Reactions” have two-way connections to the “Limbic System,” “Autonomic Nervous System,” and the “Auditory and Other Cortical Areas.”

NM hypothesizes prediction of tinnitus perception in subjects who do not have any clinical symptoms, where the limbic and autonomic nervous systems are not activated, and no reactions can be observed; abnormal neuronal activities are processed as sourceless soundwaves by the peripheral auditory system, which originate in the auditory periphery and move through the auditory pathways to the primary auditory cortex and other cortical areas. Conscious perception of the sound wave only occurs during the final cortical stages.

Additionally, tinnitus develops by the generation of abnormal neural activities in the auditory pathways. When detected by the upper-stream components of the auditory pathways, it is processed at the subconscious levels. Auditory and other cortical areas are activated and sound is consciously perceived. This conscious perception, evoked by abnormal neural activity, does not elicit any emotional or behavioral reactions other than the mere perception of sound.

Furthermore, the abnormal neural activities are evaluated subconsciously and consciously. If they are evaluated as representing a neutral event, they will not be perceived consciously. However, if the neural activities are evaluated negatively or as unknown, they will be classified as potentially unpleasant and/or dangerous which activate the limbic and autonomic nervous systems and subsequently generate negative reactions such as annoyance. Future perception of similar neural activities will receive more attention than usual and become evaluated. To create a condition of “reflex arc,” it is sufficient to experience tinnitus when at a high-level of negative emotional/autonomic state. The initial reflex arc will be created automatically. This reflex has a strong tendency to become stronger as both the signal (tinnitus) and reinforcement (reactions of the limbic and autonomic nervous systems) are contiguously present, corresponding to continuous learning that enhances the strength of the reflex.

Rauschecker et al. (2010) proposed another tinnitus model based on noise cancelation mechanism in which efferent projections from the subcallosal area are involved in the suppression of tinnitus signal as a sensory input at the thalamic level of brain processes. Functionally, the nucleus accumbens (NAc) and its correlated paralimbic circuitry were considered in the ventromedial prefrontal cortex (vmPFC), exhibiting a pivotal performance in long-term habituation to continuous unpleasant sounds.

**Figure 2:**
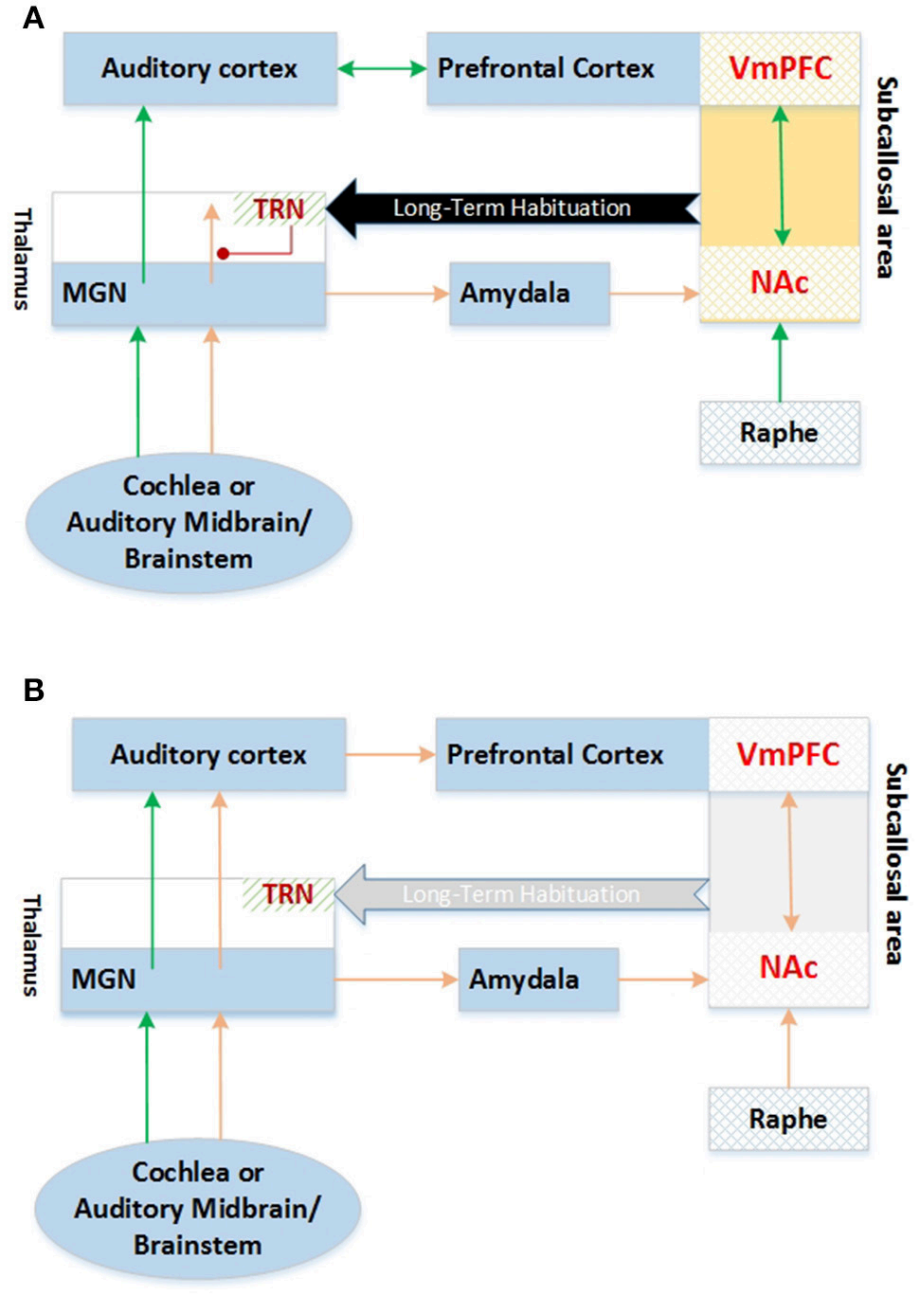
**Tune out model of Tinnitus MGN, Medial Geniculate Nucleus; TRN, Thalamus Reticular Nucleus; VmPFC, Ventromedial PreFrontal Cortex; NAc, Nucleus Accumbens (A) Compensated Tinnitus, (B) Tinnitus. Adapted from Rauschecker et al. (2010)**.

It was revealed that, in order to be perceived consciously, sound-evoked neural activity is passed from the auditory periphery through the brainstem and thalamus (MGN: medial geniculate nucleus) to the auditory cortex. For emotional content evaluation of the sound, the same signal is conducted in parallel over the amygdala to the subcallosal area (which includes the NAc region of the ventral, “limbic” striatum, and the vmPFC). The thalamic reticular nucleus (TRN) receives excitatory feedback projections from the subcallosal area. TRN consecutively applies selective inhibition at the sections of the MGN corresponding to the unpleasant sound frequencies. It was also suggested this gain-control mechanism results in a highly specific filtering (“tuning out”) of repetitive unwanted noises, which do not reach conscious perception in the auditory cortex as exhibited in Figure 2. It was recommended that if the abnormal neural activity in the peripheral system originated the sourceless tinnitus, the sound signal was being filtered out at thalamic MGN and would not be relayed to the auditory cortex in normal tuning out process. NAc-system weakening may no longer result in the tinnitus signal cancelation at the thalamic level and lead to tinnitus perception and long-term reorganization of auditory cortex, resulting in the tinnitus being carried out to the chronic phenomena. It was also implied that intermittent tinnitus might arise during the developing damage to the subcallosal area, which could strongly justify the fluctuating activity (and corresponding neurotransmitter) levels and transient filtering of the tinnitus signal.

**Figure 3:**
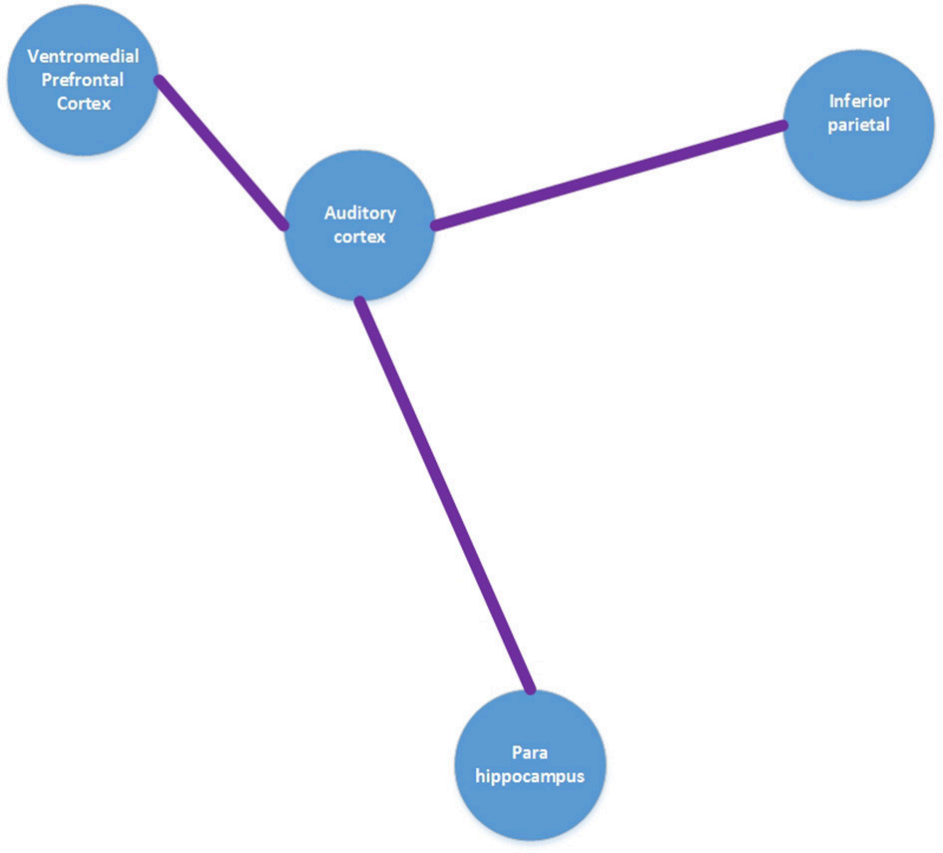
**Tinnitus core model. Adapted from De Ridder et al. (2014)**.

The integrative model of the auditory phantom perception is a recent proposal, which conceptualizes that “tinnitus core” subnetworks incorporate neurophysiological model and noise canceling process. The discussion theorizes that minimal brain areas (auditory cortex, inferior parietal area, and ventromedial prefrontal/frontopolar cortex) jointly activate to achieve the conscious perception of tinnitus. The hypothesis assumes that separable tinnitus characteristics can be extracted by evaluating resting-state magnetic and electrical studies toward the evaluation of specific characteristics and control of other parameters. Furthermore, the combination of functional neuroimaging with neuromodulation studies could provide some causal relationship between the acquired correlated networks. It was proposed that the tinnitus could be perceived as an emergent aspect of several dynamic overlapping subnetworks with different spontaneous oscillatory patterns and functional connectivity arrangement. It was theorized that communications within different subnetworks would take place in hubs, which are defined as brain regions that simultaneously participate in various brain networks and can be involved in distinct subnetworks at discrete oscillatory frequencies (De Ridder et al., 2014). The integrative model conceptualizes that the tinnitus core is comprised of the neural correlation of auditory pitch awareness and memory. Furthermore, it was hypothesized that the tinnitus core connects to other subnetworks via hubs and leads to bothersome effects such as mood disorders, distress, and lateralization. Integrative tinnitus model is illustrated in Figure 3.

It is important to note that all the aforementioned studies are limited in their scope as they have focused only on functional tinnitus-related activities of isolated regions. These studies have concentrated their efforts on structural dimensions, solo behavioral, and absolute clinical investigations. They are abstract in their descriptions and are only supported by minimal empirical evidence.

Therefore, the objective of this study is to describe a neurofunctional model of tinnitus, which can predict future results, while considering a testable framework of the structural, functional, behavioral, and clinical empirical evidence.

## Fundamental ideas and postulations of neurofunctional tinnitus model

By considering previous functional and structural neuroimaging techniques, quantitative electroencephalography (qEEG), magnetoencephalography (MEG), and animal lesion studies, distinct brain areas have been implicated in tinnitus. These areas are as follows: the peripheral auditory system, the thalamus (reticular, medial geniculate and dorsal nuclei), auditory cortex, the limbic system (anterior cingulate cortex, amygdala), brainstem (raphe nucleus), subcallosal and paralimbic areas, which include basal ganglia (ventral palladium), striatum (nucleus accumbens), and vmPFC (Ghodratitoostani et al., 2016). Figure 3 provides a schematic tinnitus network overview of different brain areas as composed by integrating data from SPECT, PET, fMRI and MEG studies research in tinnitus.

**Figure 4:**
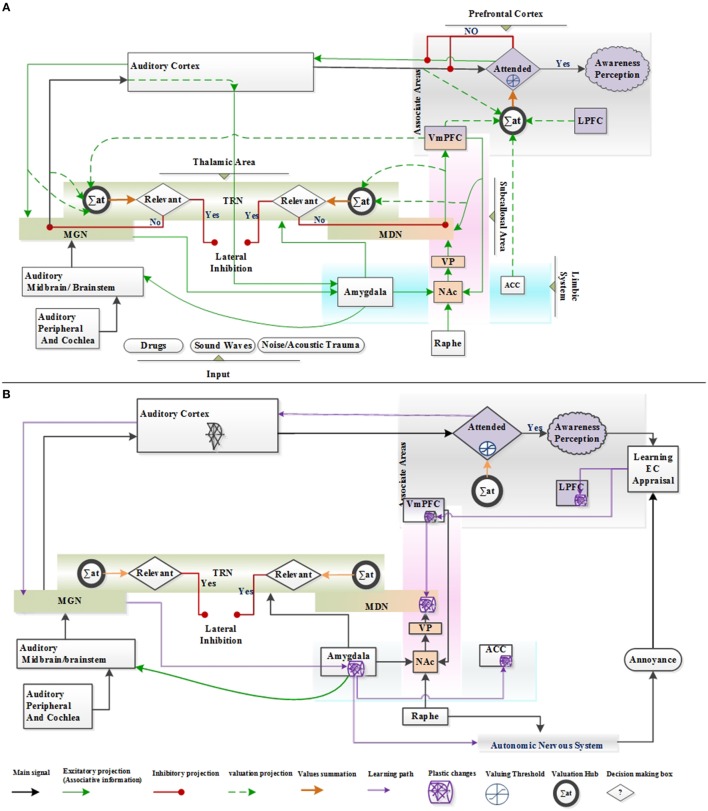
**Anatomical structure of the proposed Neurofunctional Tinnitus Model; LPFC, Lateral prefrontal cortex; vmPFC, ventromedial prefrontal cortex; NAc, nucleus accumbens; ACC, anterior cingulate cortex; MGN, medial geniculate nucleus; TRN, thalamic reticular nucleus; VP, ventral palladium; MDN, medial dorsal nucleus; Basal ganglia include VP and NAc; EC, Evaluation Conditioning. (A)** Phantom sound perception without negative reaction is defined as “Neutral stage” within the Neurofunctional Tinnitus Model; **(B)** The evaluation conditioning and appraisal learning procedure and anatomical part of plastic change in tinnitus brain network, is identified as “Clinical Distress stage” of the Neurofunctional Tinnitus Model.

Authors agree that the continuous or intermittent abnormal neuronal activity at the peripheral (Kaltenbach et al., 1998; Brozoski et al., 2002; Chang et al., 2002), midbrain (Jastreboff and Sasaki, 1986; Chen and Jastreboff, 1995), auditory paths (Kaltenbach and McCaslin, 1996; Zhang and Kaltenbach, 1998; Eggermont and Komiya, 2000; Noreña and Eggermont, 2003; Seki and Eggermont, 2003; Zhang et al., 2011)or associative cortices such as limbic area (Zikopoulos and Barbas, 2007; Yu et al., 2009; Kaping et al., 2011; Weinberger, 2011; Kuchinke et al., 2015) can cause phantom sound generation. This aberrant neural activity can be generated by any type of acoustic traumas, aging, brain lesions, and medicine. The Neurofunctional Tinnitus Model hypothesizes that the perception of sound fundamentally depends on the allocation of attentional resources via frontal cortex, which in turn, depends on the cognitive-emotional value and the relevance of the phantom stimulus to the context. Small value of stimuli has less chance to allocate attentional resources. Noise-canceling mechanisms at the thalamic MGN level, which are governed by TRN inhibitory projections, maintain the weakness of the upward irrelevant signal. However, high cognitive-emotional value of sensory stimuli may allocate adequate attentional resources and trigger a top-down suppression process that acts on the thalamus noise-canceling mechanism and may lead to the awareness perception of the tinnitus phantom sound. Initially, the phantom sound is considered neutral, which we have defined as the “Neutral stage” within the Neurofunctional Tinnitus Model as illustrated in Figure 4A.

Authors hypothesize that the malfunction of the noise canceling mechanism reinforces higher rates of the phantom sound that reaches the auditory cortex and consequently facilitates the abnormal neural plasticity in auditory cortex and tonotopic reorganization coined as “Central tinnitus.” Centralization can develop during both neutral and clinical stages of the Neurofunctional Tinnitus Model.

The patient's general suspicion can result in negative cognitive interpretation. This negative appraisal and evaluative conditioning (EC) learning mechanism, jointly enhance the associated cognitive-emotional value and the persistence of the sound conscious (attended) awareness perception. Therefore, the probability of limbic system involvement and the appearance of negative cognitive-emotional reactions increase (such as attention deficit, anxiousness, insomnia, and phobias). If the cognitive-emotional value remains at low levels, tinnitus will cease or be perceived as the failure of noise canceling mechanism and suppression at the thalamic level. This condition is identified as “Clinical Distress stage” of Neurofunctional Tinnitus Model as exhibited in Figure 4B.

It is postulated that rapidly encoded tinnitus, such as acoustic trauma tinnitus-induced, relates to temporary interactions between the hippocampus and auditory neocortical processing regions. Due to complexity reduction, we ignore this extraordinary transient hippocampus involvement in Neurofunctional Tinnitus Model.

### Model compartments

#### Auditory peripheral and cochlear

This includes external-middle ears organs and cochlea, which receive acoustical pressure and sound as input and propagate electrical signals from auditory nerves (Ghodratitoostani et al., 2016).

#### Auditory midbrain/brainstem

This includes cochlear nucleus, superior olivary complex (SOC), inferior colliculus auditory pathway, and raphe nucleus. Cochlear nucleus sends projection to SOC and inferior colliculus and receives input from, auditory nerve which encompasses cochlea (Kraus and Canlon, 2012; Ghodratitoostani et al., 2016). The SOC receives inputs from the cochlear nucleus and relays the signal to inferior colliculus (Kraus and Canlon, 2012; Ghodratitoostani et al., 2016). We have ignored the internal parts of the auditory midbrain/brainstem compartment to decrease the complexity of the model.

#### Raphe nucleus

The serotonin originates in the raphe nucleus of the cerebellum. Its non-equilibrium can disturb normal sleep (Ghodratitoostani et al., 2016).

#### Thalamic area

It is located between cerebral cortex and midbrain. Despite the several sections of the thalamic area, we have proposed that medial geniculate nucleus (MGN), mediodorsal nucleus (MDN), and TRN play crucial roles in tinnitus generation. The **MGN** acts as a thalamus relay in the auditory system that receives excitatory inputs from auditory midbrain (IC), auditory cortex, and inhibitory inputs from TRN. In return, it sends excitatory projections into TRN, auditory cortex, and amygdala. The **MDN** sends excitatory output to TRN and vmPFC and receives excitatory projections from vmPFC and ventral palladium of the subcallosal area. **TRN** receives excitatory inputs from MGN, auditory cortex, MDN, vmPFC and amygdale. It sends inhibitory projections to thalamus MGN and MDN (Ghodratitoostani et al., 2016).

#### Auditory cortex

It includes primary and secondary and associative auditory cortices, which receive excitatory inputs from MGN and send excitatory projections to MGN, TRN, prefrontal cortex, and amygdala (Ghodratitoostani et al., 2016).

#### Subcallosal area

It includes nucleus accumbens (**NAc**), ventral palladium (**VP**), and **vmPFC**. NAc receives excitatory inputs from the amygdala. The vmPFC accepts serotonergic inputs from raphe nucleus, which trigger VP via unidirectional excitatory projection. VP receives excitatory input from NAc and sends excitatory projection to MDN (Ghodratitoostani et al., 2016).

#### Prefrontal cortex

It includes lateral prefrontal cortex (**lPFC**), ventromedial prefrontal cortex (vmPFC, and associative cortex in which lPFC and vmPFC send excitatory outputs into the valuation hub of attentional allocation as a cognitive-emotional value. The vmPFC sends excitatory projections to TRN, MGN, MDN, and NAc (Ghodratitoostani et al., 2016).

#### Limbic system

It includes amygdala and anterior cingulate cortex (**ACC**). The ACC sends output to the valuation hub as a purely emotional value of familiar known audio signals. The amygdala sends excitatory projections into TRN, midbrain (IC) and NAc. The NAc projection may trigger vmPFC via VP of the subcallosal area. Amygdala receives excitatory projections from MGN and auditory cortex (Ghodratitoostani et al., 2016).

### Cognitive processes involved in initiating tinnitus “Neutral stage,” noise canceling mechanism, and lateral inhibition circuitry

The bottom-up selective attention processes support the suppression of irrelevant stimuli, which may occur at the early stages by the TRN along the “lateral inhibition mechanism” (Kiang et al., 1970; Tyler, 2006; Zikopoulos and Barbas, 2012). The thalamocortical neurons, carrying the relevant stimuli up from thalamus to cortex via TRN, excite the adjacent TRN neurons which in turn inhibit the irrelevant thalamic cortical neuron carriers. These adjacent TRN neurons, inhibit the TRN neurons connected to the relevant thalamocortical carriers leading to dis-inhibition of the relevant signal carriers (Pinault and Deschênes, 1998). This mechanism of lateral inhibition ideally suppresses the noise originated from distracters and facilitates the processing of important stimuli. The amygdala, posterior orbito frontal cortex (pOFC), and mediodorsal (MD) thalamus ending at the TRN may suppress the signal of distracting stimuli at sensorial cortices (Zikopoulos and Barbas, 2007). TRN can perform gain–control function of the thalamo-cortical transmission in a highly localized manner. Due to serotonergic neurons in the subcallosal area, TRN inputs vigorously inhibit MGN neurons in the conscious state and anesthetic trials in a specific high frequency manner (Yu et al., 2009). Thalamus projections to TRN can modulate transmission from the sensory periphery and brainstem to the cerebral cortex (Yu et al., 2009).

Furthermore, voxel-based morphometric tinnitus patient studies revealed the reduction in gray matter in vmPFC that resulted in the decline of the vmPFC inhibitory output, leading to increased activity of NAc (Schlee et al., 2009). While auditory cortical activity is essential for conscious perception of phantom sound, NAc-TRN in Neurofunctional Tinnitus Model is postulated as noise canceling mechanism for preventing the unpleasant permanent sound to reach auditory cortices. This mechanism is in partial agreement with the tuned out model of tinnitus (Rauschecker et al., 2010).

The amygdala is a crucial component of noise canceling circuitry for processing sensory inputs with emotional value, which is related to both the mediodorsal thalamus nucleus (MD) and the orbitofrontal cortex (Ghashghaei et al., 2007).

#### Valuation process

Selective attention prioritizes the processing of behaviorally relevant stimuli at the expense of processing of irrelevant stimuli (Tsotsos, 2011). Relevance of stimulus requires an active associated neuronal network to indicate its related value or its reward outcome in a special context (Kaping et al., 2011). Recent evidence proposes that the brain network regularly determines and processes the values related to the stimuli that effectively bias the attentional stimulus selection against the more valuable stimuli in the peripherals (Shuler and Bear, 2006; Seitz et al., 2009; Anderson et al., 2011). Neural clusters related to valuations within the vmPFC were disassociated from the top-down goals network in spatial attentions. Behavioral analysis suggested that shifting attention to less important stimuli required specific mechanism to overcome a motivational bias of attending to the more important stimuli (Anderson et al., 2011).

In a recent paper, the visual valuation hub demonstrated that the value-selection response correlated with the activity of neurons located across the medial to lateral extent of the PFC (vmPFC), the anterior cingulate cortex (ACC), and the lateral PFC (Kaping et al., 2011). The highly valued stimuli suppression in human and related macaque studies have been conceptualized as a “self-control” process (Kaping et al., 2011), which is associated with the alterations in neural activity level of the dorsolateral PFC and the rostral ACC in human subjects (Hare et al., 2009). Together, these findings propose that the valuation hub plays a similar role across different sensory modalities and that the same process within the auditory stimuli can drive attentional resources to hear tinnitus in the Neurofunctional Tinnitus Model.

#### Conscious awareness perception processes and attention

The ability to consciously report sensory inputs is theorized as the perception process. We propose to add more details to the model of distinction between awareness and attention (Lamme, 2003; Watanabe et al., 2011). Conscious inputs originate from triggers in sensory pathways or can be retrieved from cortices and memory.

**Figure 5:**
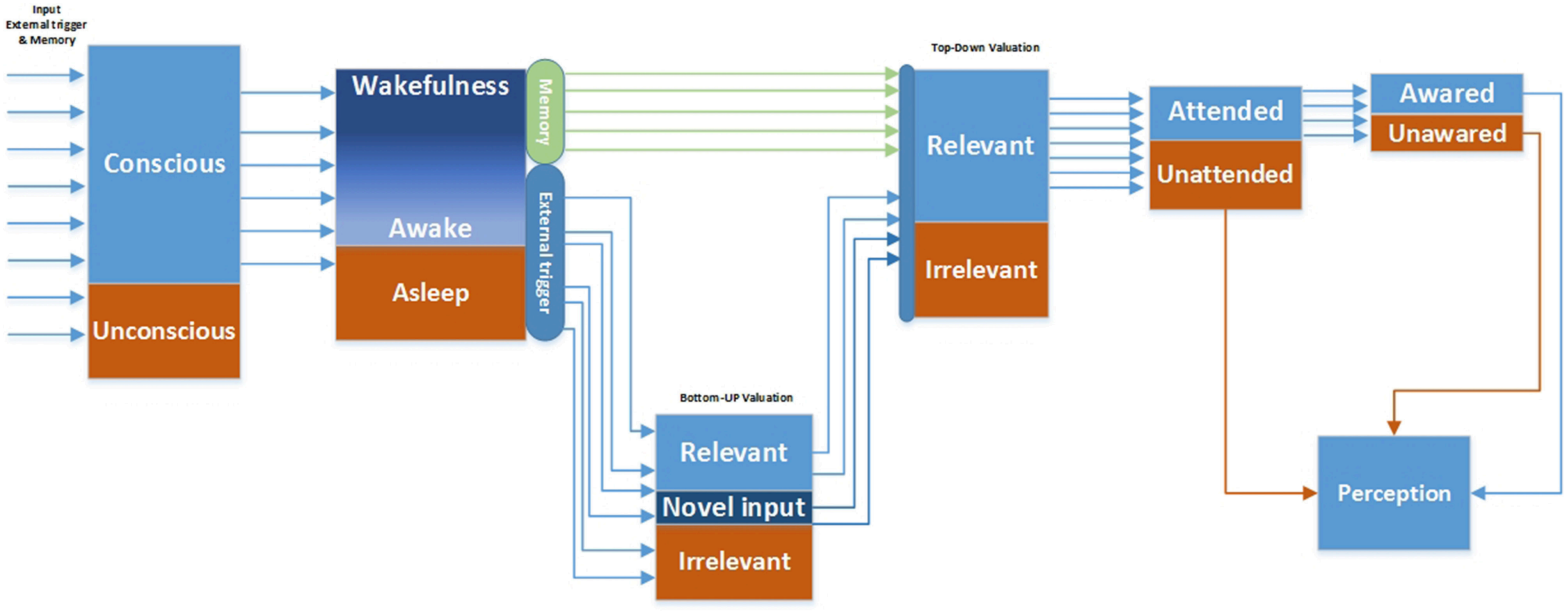
**Conscious perception process model**.

The proposed Conscious perception process (CPP) conceptual model provides logical solutions as depicted in Figure 5. It discriminates the inputs in conscious and unconscious states in the initial stages. In this approach, the consciousness level alters from deep sleep and reaches wakefulness. The wakefulness levels dynamically fluctuate during awakeness.

The triggered inputs, coming through sensory pathways, are evaluated via automated bottom-up valuation processes. The relevant and novel signals along with pulled memory signals reach the top-down valuation process stage. The top-down relevant (cognitive emotional valued) signal is permitted to reach the attentive processes whereby the signal can become aware or unaware (Graziano and Webb, 2015).

According to the CPP conceptual framework, conscious perception can be characterized into unattended, attended, and attended awareness. Attention represents a cognitive mechanism that allows certain information to be processed more intensively. Attention facilitates the transmission of the selected information across the cortex in comparison to the non-attended information (Cohen et al., 2012). Conscious awareness requires attention because it permits information to remain on-line long enough to be completely processed by an expanded network of cortical associations (Cohen et al., 2012). Structurally, the lateral prefrontal cortex (LPFC) is associated with attention and inhibitory control (Sasaki et al., 1989; MacDonald et al., 2000; Ploner et al., 2005), particularly in auditory gating (Woods and Knight, 1986; Knight et al., 1989).

By considering the Neurofunctional Tinnitus Model and the CPP, we proposed that the clinical distress tinnitus could only be developed and maintained during conscious (attended) awareness perception of neutral tinnitus. Several studies have reported no tinnitus-related neural activity in patients during coma, anesthetics, vegetative states, restless eye movement (REM) sleep, and dreams (De Ridder et al., 2014).

Furthermore, the top-down attention and conscious awareness processes suggest that the triggered inputs and phantom sound cannot be perceived without attended awareness. This finding is in agreement with the unconditional sensitization model, which proposed that the highly complex auditory tinnitus stimulus pattern is controlled by attention (Zenner et al., 2006).

### Cognitive processes involved in the centralization of tinnitus

#### Acoustical memory for repeated items

It was recently proposed that the memory systems should not be classified in association with the conscious states as the conscious states would not influence the encoding or retrieval of information (Henke, 2010). The critical factors seemed to be the quantity of elements required to encode the number and the duration of information presentations.

Rapidly encoded novel associations are postulated to relate interactions between the hippocampus and certain neocortical processing sites. In temporally extended learning state, the hippocampus might temporarily improve learning performance, but this is not essential for successful information retention, consolidation or retrieval. The memory system relies heavily on the interactions between neocortical structures, the basal ganglia, the cerebellum, and the parahippocampal gyrus (Henke, 2010; Hannula and Greene, 2012).

Considering the Henke model, abnormal neural activity or sound perception best fit the temporally extended learning system as related to tinnitus characteristic. The hippocampus structure is not necessarily involved in tinnitus generation; however, the paralimbic is involved in the generation of tinnitus. This proposal is supported by the results of a recent resting-state connectivity network fMRI study (Maudoux et al., 2012) which found no correlation between the hippocampus and resting-state network activity. However, it found a significant correlation between typical tinnitus connectivity networks and the parahippocampal, cerebellum, basal ganglia, subcallosal region, thalamic areas, and amygdala regions. It also exhibited a significant correlation between the anterior cingulate, auditory and prefrontal cortices (Maudoux et al., 2012).

These two studies suggest that clinical distress tinnitus is more prone to emerge from a gradual learning procedure rather than a single exposure to an external stimulus.

### Cognitive processes in clinical distress stage

#### Cognitive-emotional appraisal

The cognitive-emotional appraisal is a mechanism that emerges as a reaction to the differences between the information stored in memory and the actual information. Generally, patients have no intuitive understanding of tinnitus signal; thus, the risk of negative appraisal will increase. One of the crucial causes of appraisal is the patient's hypochondriac impression of neutral phenomena (de Maddalena and Pfrang, 1993a,b; Marciano et al., 2003). Since, the individual's response to illness is mainly shaped by their understanding of the illness, the personal disorder concept becomes an important factor (Zenner et al., 2006). Examples for such personality disorders include the potential triggers of tinnitus and available tinnitus managements and treatments leading to appraisals such as “tinnitus is detrimental to my health” or “now, I am becoming deaf” (Zenner et al., 2006).

#### Evaluation conditioning (EC)

The Neurofunctional Tinnitus Model hypothesizes that negative symptoms related to tinnitus emerge in the neutral tinnitus stage together with the EC learning procedure. When presented in association with a negative or positive unconditioned stimulus (US), EC points to the change in the valence of the cognitive-emotionally neutral conditioned stimulus (CS). This change in the valence is the retained response to the previously neutral stimulus (De Houwer et al., 2001; Bar-Anan et al., 2010). Recently, studies have demonstrated that attention as the stimulus focus does not cause EC; rather, to promote the acquisition of contingency awareness, the attention to the contingencies between stimuli seem to be crucial in EC (Stahl et al., 2009; Kattner, 2012; Hütter and Sweldens, 2013). Furthermore, the ERP-EEG source localization indicated a role of medial-frontal brain regions as the likely origin of early valence discrimination signals (Kuchinke et al., 2015). The prevailing tinnitus models emphasize classical conditioning learning via pairing of the external events with the causal physical events.

We project that the Clinical Distress tinnitus stage is developed and maintained through the EC learning only in conscious (attended) awareness perception of CS (neutral tinnitus) and US (comorbidities reaction) contingency. EC encodes the memory through the learning pathway, Figure 4B. Encoding memory initiates plasticity in VMPFC and LPFC areas of frontal cortex which result in plasticity in several regions of the Limbic and Auditory systems via the subcallosal area and the corticothalamus auditory pathway.

In the subcallosal area pathway, the EC encoded memory consolidates in MDN to bias tinnitus valence of the automated bottom-up valuation process. Intermittent occurrences of EC learning reinforce the tinnitus valence. In the corticothalamus auditory pathway, the EC encoded memory leads to the plasticity of the MGN and lateral nucleus of amygdala (LA). LA connects to the central nucleus of the amygdala (CE) directly and via other amygdala regions. The output of the CE creates plasticity in ACC and controls the expression of the distress responses (such as fear), the related autonomic nervous system (e.g., blood pressure and heart rate), and endocrine (pituitary adrenal hormones) responses (Weinberger, 2011).

Pavlovian conditioning (PC) learning occurrence needs adequate frequency of pairing of the CS and US to encode the memory. Violation in synchrony of CS and US weakens the encoded memory. In return, EC learning (valence) only happens when pairing of the US and CS also incorporates awareness of contingency. Furthermore, unpaired occurrences of CS or US have no effects on valence. Therefore, we argue that EC learning is the only well-known mechanism which can explain tinnitus clinical distress development and maintenance.

### Model function and information flow

In this Neurofunctional Tinnitus Model, we proposed that in parallel with centralization, during the two stages of tinnitus development, phantom sound can lead to clinical symptoms. Initially, continuous or intermittent abnormal neuronal activities are developed in the peripheral or midbrain auditory paths or associative cortices (such as the limbic area). Several other factors such as different types of lesions, acoustic trauma, and drugs can also cause temporary abnormal activity in the auditory cortices. Depending on the availability of attentional resources, the phantom sound may be suppressed or perceived. Weaker cognitive emotional value of this potential phantom sound lowers the chances of its conscious (attended) awareness perception.

Irrelevance of the cognitive-emotional evaluations biases low valuation scores. This biasing of cognitive-emotional valuation strengthens the suppressive effects of both the lateral-inhibition of the bottom-up selective attention and the noise canceling mechanisms at the MGN thalamic nucleus. The noise canceling mechanism, governed by TRN inhibitory projections, reduces the frequency of phantom sound reaching the auditory cortex and maintains irrelevancy of the signal evaluation.

Based on the EC learning procedure and cognitive appraisal, phantom sound perception during Neutral tinnitus stage, gradually strengthens the negative valence of perceived tinnitus, and interprets it as suspicious and/or indicative of a disease. The cortical top-down processes weaken the noise canceling effects (Rauschecker et al., 2010). The phantom sound is considered a relevant stimulus which results in gradual formation of a vigilant auditory expectation of neutral tinnitus perception. The consequences are engendering the sense of cognitive and emotional reactions, which usually leads to negative reactions such as stress and depression (Halford and Anderson, 1991; Robinson, 2007; Robinson et al., 2007), anxiousness (Halford and Anderson, 1991; Langguth, 2011), hypochondriasis (Marciano et al., 2003), phobias (Zenner et al., 2006; Adjamian et al., 2009), and insomnia. These cognitive and emotional reactions, and related comorbidities, may cause tinnitus or tinnitus may cause these conditions. Ultimately, via the EC learning procedure, clinical distress tinnitus can be caused by the contingent relationship between the perceived relevant sound and negative valance of cognitive and emotional reactions.

Failure of lateral inhibition, caused by the effects of drugs, somatosensory modulation or other external negative events creates neuronal disturbances within the GABAergic pathways. These disturbances can cause abnormal activity where sourceless sounds may frequently reach the auditory cortex. However, regardless of the development of Clinical Distress stage from the conscious (attended) awareness perception of the neutral phantom sound, the neuroplasticity and long-term associative memory consolidation forms in the auditory cortices. Depending on the availability of attentional resources, the phantom sound may be perceived. From this point on, we name the conscious perception of phantom sound as the neutral/clinical distress tinnitus. Furthermore, memory consolidation during centralization can bypass the noise canceling procedure, which strengthens the irrelevant cognitive-emotional value of the bottom-up attention. This leads to hyperactivity of noise canceling procedure, which is in agreement with the reduction of subcallosal gray matter volume. Correspondingly, the failure of noise canceling process is recognized in the amygdala evaluation procedure. The basolateral part of the amygdala receives excitatory projections from both MGN and auditory cortex which can trigger TRN to apply more inhibitory force for irrelevant stimuli. Persistence of abnormal activity in auditory peripheral is not necessary to the perception of tinnitus; however, it can reinforce neuroplasticity and associative memory.

## Neurofunctional tinnitus model predictions and discussions

We hypothesize that conscious (attended) awareness perception is necessary for neutral tinnitus to turn to clinical distress tinnitus. This concept has not been disclosed in prior tinnitus models. In our opinion, unattended phantom sound or neutral tinnitus cannot cause bothersome or distress symptoms. The attended awareness to sourceless sound, allows information to remain on-line long enough to be thoroughly processed by a distributed network of cortical circuits, the limbic and autonomous nervous system.

Zenner et al. (2006) proposed a role for auditory attention in establishing the neural changes underlying tinnitus, although a specific mechanism for attention and the circumstances leading to its engagement were not described (Zenner et al., 2006). The Neurofunctional Tinnitus Model asserts that in order to perceive tinnitus, the valuation process in frontal cortex plays a crucial role in the prioritization of sensory inputs in attention resource allocation and lead to clinical distress tinnitus development. The three stages involved are: tinnitus generation, maintenance, and clinical distress development.

1. Tinnitus Generation: Our hypotheses regarding noise canceling mechanism failure is in agreement with the Winkler et al. (2009) model, which argues that, attention is considered to be a factor in modulating the detection of prediction failure (detection of deviance) and in promoting the stimulus-driven binding of sensory attributes to create new auditory objects in dynamic auditory environments. We further propose that such failure results in the perception of neutral phantom sound, which is not mentioned in Rauschecker's tuning out noise model (Winkler et al., 2009; Rauschecker et al., 2010; Roberts et al., 2013).
2. Tinnitus Maintenance: We argue that the auditory attention involvement (Roberts et al., 2013) and the discrepancy between top-down and bottom-up attentional processes can bias the cognitive-emotional value of the neutral phantom sound. We also hypothesize that the valuation hub in pre-frontal cortex continuously regulates the persistence of tinnitus perception and compares the value of intermittent attended awareness perceived tinnitus (phantom sound) with all sensory and auditory inputs. During conscious (attended) awareness of tinnitus, according to the patient's emotional stability, appraisal magnifies the cognitive-emotional value of tinnitus and results in increased duration of the perception. Furthermore, independent of patient perception, the continuous, and repetitive abnormal neural activities reaching the auditory cortex, form plasticity in auditory pathways and lead to the auditory memory of tinnitus “centralized tinnitus.” This is in agreement with the Henke memory model (Henke, 2010; Roberts et al., 2013).
3. Tinnitus Clinical Distress: We propose that cognitive-emotional cognitive value of tinnitus increases via the negative appraisal and evaluative conditional learning mechanisms during conscious (attended) awareness perception of tinnitus. The tinnitus signal links with the limbic system and actuates annoyance, leading to clinical distress. We also agree that the conscious awareness of tinnitus can be suppressed and substantially modulated when the patients engage in cognitively demanding tasks (Searchfield et al., 2007; Searchfield and Kobayashi, 2012).

Due to the lack of contingency and pairing CS and US, the EC learning vs. conventional Pavlovian conditioning (PC) can strongly legitimize the emergence of various tinnitus symptoms in patients. However, to develop learning, both PC and EC are needed to simultaneously perceive CS (tinnitus) and US. This seems to be in violation of assumptions of the neurophysiological tinnitus model (Jastreboff, 1990). We propose that, not only conscious perception of tinnitus, but also conscious (attended) awareness perception of tinnitus contingency and US (distress symptom causes) are needed to develop, maintain, and rehabilitate tinnitus.

According to the Neurofunctional Tinnitus Model, the patients can be categorized into two general groups of neutral and clinical distress as summarized in Table 1. In the neutral stage, we have divided the patients into those who only perceive tinnitus (Type NT-I), those who perceive tinnitus with auditory cortex plasticity (Type NT-II), and those who do not perceive tinnitus and have no auditory cortex plasticity (Type NT-X). Since, biasing of their negative valence via EC may support future prevention programs, type X patients, who no longer perceive tinnitus, can become potential future tinnitus patients. The patients in the clinical stage can be further divided into two groups: those who perceive tinnitus and only have limbic system plasticity (Type CL-I) and those who have Type CL-I symptoms and auditory cortex plastic changes (Type CL-2). This novel view may help us investigate corresponding diagnostic assessment results in correct classifications and lead to the selection of the most appropriate rehabilitative methodologies.

**Table 1:** **Tinnitus patient categorization accordance to the Neurofunctional Tinnitus Model development stage; Y, Yes and N, No**.

**Tinnitus Stage**	**Type**	**Phantom sound perception**	**Negative reaction**	**Plasticity in Auditory cortex**	**Plasticity in Limbic system**
Neutral	NT-I	Y	N	N	N
	NT-II	Y	N	Y	N
	NT-X	N	N	N	N
Clinical Distress	CL-I	Y	Y	N	Y
	CL-II	Y	Y	Y	Y

Further experimental and clinical studies on tinnitus brain mapping, brain imaging, neuromodulation, cognitive behavioral therapy, and head modeling to evaluate and validate the following Neurofunctional Tinnitus Model predictions and suggested validation methodologies:

- In cognitive rehabilitation approaches, rehabilitation cannot happen without conscious (attended) awareness perception of tinnitus
  ◦ Examining changes in clinical distress symptoms during unconscious vs. conscious and conscious attended cognitive intervention. Recently, Probst et al. (2016) described a model examining emotional states, the perceived loudness of tinnitus, and tinnitus distress in patients who are in the clinical distress stage and not in the developing stage (Probst et al., 2016). However, if we consider the clinical distress as a dynamic spectrum to maintain tinnitus bothersome, the results support our hypothesis that, consciousness is necessary for neutral tinnitus to turn to clinical distress tinnitus. In addition, the tinnitus bothersome (distress) fluctuations occur during conscious (attended) awareness perception [ability to cognitively report scale of his/her tinnitus]. Furthermore, in the Probst et al. (2016) study, the patients scaled their stress levels [awareness of contingency with unconditional stimulus]. We agree that the awareness of contingency and conditional stimuli is necessary for EC learning procedure but not in classical conditioning learning.
- Tinnitus psychoacoustic specifications encode to memory in auditory cortex during centralization of tinnitus
  ◦ Use resting-state fMRI-EEG analysis to investigate the brain network activity and connectivity in correlation with loudness and pitch before and after electrical neuromodulation stimulation trials in auditory cortex.
- Continuous evaluation of Tinnitus valence is performed in PFC
  ◦ Use electrical neuromodulation stimulation on dorsolateral prefrontal cortex to indicate decrease in bothersome and negative valence.
- Change in tinnitus valence leads to change in attentional allocation and duration of conscious perception
  ◦ Perform cognitive behavioral therapy to prove that decreasing the tinnitus negative valence can decrease frequency of tinnitus perception and bothersome.
  ◦ Perform cognitive counseling trials to improve patient knowledge of tinnitus, which can decrease cognitive-emotional appraisal, lead to decrease in negative valence, and decrease frequency of tinnitus perception and bothersome.
- Cognitive disorders (like insomnia, stress) cannot generate tinnitus but, they can develop tinnitus negative valence and bothersome
  ◦ Perform clinical trials and meta-analysis on patients with cognitive disorders.
- Conscious pairing of adequate pleasant audio-visual stimulus with tinnitus can decrease tinnitus negative valence
  ◦ Perform pleasant multi-modality virtual reality application in future trials.

## Author contributions

IG, Principle investigator. YZ, Cognitive Neuroscience adviser. AD, information flow adviser. SS, Audiology adviser. HE, Neuroscience adviser. TS, Otolaryngology and clinical adviser.

### Conflict of interest statement

The authors declare that the research was conducted in the absence of any commercial or financial relationships that could be construed as a potential conflict of interest.
